# Synergy and competition during the anaerobic degradation of N-acetylglucosamine in a methane-emitting, subarctic, pH-neutral fen

**DOI:** 10.3389/fmicb.2024.1428517

**Published:** 2024-12-11

**Authors:** Katharina Kujala, Oliver Schmidt, Marcus A. Horn

**Affiliations:** ^1^Water, Energy and Environmental Engineering Research Unit, University of Oulu, Oulu, Finland; ^2^Department of Arctic and Marine Biology, UiT The Arctic University of Norway, Tromsø, Norway; ^3^Institute of Microbiology, Leibniz University Hannover, Hannover, Germany

**Keywords:** N-acetylglucosamine, intermediary ecosystem metabolisms, anaerobic feed chain, intermediates, methyl-CoM-reductase genes, hydrogenase genes, microbial community

## Abstract

Peatlands are invaluable but threatened ecosystems that store huge amounts of organic carbon globally and emit the greenhouse gasses carbon dioxide (CO_2_) and methane (CH_4_). Trophic interactions of microbial groups essential for methanogenesis are poorly understood in such systems, despite their importance. Thus, the present study aimed at unraveling trophic interactions between fermenters and methanogens in a nitrogen-limited, subarctic, pH-neutral fen. *In situ* CH_4_ emission measurements indicated that the fen is a source of CH_4_, and that CH_4_ emissions were higher in plots supplemented with ammonium compared to unsupplemented plots. The amino sugar N-acetylglucosamine was chosen as model substrate for peat fermenters since it can serve as organic carbon and nitrogen source and is a monomer of chitin and peptidoglycan, two abundant biopolymers in the fen. Supplemental N-acetylglucosamine was fermented to acetate, ethanol, formate, and CO_2_ during the initial incubation of anoxic peat soil microcosms without preincubation. Subsequently, ethanol and formate were converted to acetate and CH_4_. When methanogenesis was inhibited by bromoethanesulfonate, acetate and propionate accumulated. Long-term preincubation considerably increased CH_4_ production in unsupplemented microcosms and microcosms supplemented with methanogenic substrates. Supplemental H_2_-CO_2_ and formate stimulated methanogenesis the most, whereas acetate had an intermediary and methanol a minor stimulatory effect on methane production in preincubated microcosms. Activity of acetogens was suggested by net acetate production in microcosms supplemented with H_2_-CO_2_, formate, and methanol. Microbial community analysis of field fresh soil indicated the presence of many physiologically unresolved bacterial taxa, but also known primary and secondary fermenters, acetogens, iron reducers, sulfate reducers, and hydrogenotrophic methanogens (predominately *Methanocellaceae* and *Methanoregulaceae*). Aceticlastic methanogens were either not abundant (*Methanosarcinaceae*) or could not be detected due to limited coverage of the used primers (*Methanotrichaceae*). The collective results indicate a complex interplay of synergy and competition between fermenters, methanogens, acetogens, and potentially iron as well as sulfate reducers. While acetate derived from fermentation or acetogenesis in this pH-neutral fen likely plays a crucial role as carbon source for the predominant hydrogenotrophic methanogens, it remains to be resolved whether acetate is also converted to CH_4_ via aceticlastic methanogenesis and/or syntrophic acetate oxidation coupled to hydrogenotrophic methanogenesis.

## Introduction

Arctic and boreal peatlands are long-term carbon stores which have sequestered carbon dioxide (CO_2_) over millennia, and as a consequence have accumulated thick organic layers that can span several meters (Tarnocai et al., 2009). Northern peatlands are considered especially affected by climate warming and may switch from net C sinks to net C sources if greenhouse gas (methane (CH_4_) and CO_2_) emissions exceed sequestration with climate warming and changes in precipitation (Gallego-Sala et al., 2018). Moreover, peatland carbon may be lost by leaching of organic matter into streams, lakes, and nearshore ocean systems that may substantially affect water quality and nutrient regimes of typically nutrient-limited aquatic systems in the Arctic (Limpens et al., 2008).

Microorganisms are drivers of greenhouse gas production and consumption in peatland ecosystems (Conrad, 1996; Kolb and Horn, 2012), and microbial activity thus determines whether peatlands are net sources or sinks for greenhouse gasses. In water-saturated anoxic peat with low availability of alternative electron acceptors such as nitrate or sulfate, mineralization of organic matter will be driven primarily by fermentations and methanogenesis (Limpens et al., 2008; Drake et al., 2009). Carbohydrate monomers like glucose, xylose, and N-acetylglucosamine (NAG), which are derived from complex polymers such as cellulose, hemicellulose, and chitin, respectively, are degraded via primary and secondary fermentations to alcohols, short chain fatty acids like acetate, formate or propionate as well as CO_2_ and molecular hydrogen (H_2_) (Drake et al., 2009). These fermentation products are eventually consumed by methanogens, which form CH_4_ and CO_2_ (Drake et al., 2009).

Chitin (a component of fungal cell walls and the exoskeleton of many invertebrates) is the second most abundant biopolymer on earth, and together with ligno-cellulose, the two refractory compounds constitute the main source of organic matter in wetlands (Mann, 1988; Mitsch, 1994). NAG is the single monomer of chitin, and it has been shown for anoxic river sediments that chitin-derived NAG is typically consumed by chitinolytic fermenters although many non-chitinolytic fermenters have the ability to degrade NAG as well under the premise that it becomes bioavailable (Gooday, 1994; Wörner and Pester, 2019). NAG is also a major monomer in peptidoglycan, a component of bacterial cell walls, and since dead microbial cells constitute an abundant and readily degradable source of organic carbon in peatlands, peptidoglycan-derived NAG can be assumed to be an important substrate for non-chitinolytic fermenters in these ecosystems (Pazos and Peters, 2019; Tveit et al., 2015).

Since NAG is an amino sugar, the decomposition of NAG-containing polymers from organic matter could be important for both carbon and nitrogen cycling in peatlands (Kang et al., 2005; Beier and Bertilsson, 2013). However, information on how NAG is fermented and what trophic links between NAG-consuming fermenters and methanogens exist in peatlands is scarce (Wüst et al., 2009).

Communities of methanogens in peatlands are typically dominated by hydrogenotrophic methanogens that use H_2_ (and often also formate) as electron donor to reduce CO_2_ to CH_4_ and/or aceticlastic methanogens that split acetate into CH_4_ and CO_2_ (Kotsyurbenko et al., 2019; Bräuer et al., 2020). Methanol disproportionation to CH_4_ and CO_2_ or complete reduction to CH_4_ with H_2_ as electron donor are well known. Some peat methanogens utilize methanol, but it is considered to be less important for the overall CH_4_ production in peatlands (Conrad, 2020). The methanogenic potentials and the contribution of hydrogenotrophic versus aceticlastic methanogenesis in peatlands are variable and depend on various factors such as temperature, pH, hydrology, and vegetation (Kotsyurbenko et al., 2007; Rooney-Varga et al., 2007; Hines et al., 2008; Conrad, 2020). As an example, the deposition of easily degradable organic carbon compounds from *Carex* roots especially during the growing season has been conceptualized to stimulate methanogenesis and favor aceticlastic methanogens in fens whereas hydrogenotrophic methanogenesis is often more important in acidic bogs covered predominately by *Sphagnum* mosses that generally produce less methane (Kelly et al., 1992; Kotsyurbenko et al., 2019). Furthermore, CH_4_ production and the methanogenic communities in one peatland often change seasonally, with depth (i.e., the recalcitrance of the peat organic matter), and based on the microtopography of the sampling site (Galand et al., 2003; Reiche et al., 2008; Hodgkins et al., 2014; Zalman et al., 2018).

In the present study, NAG was chosen as an easily degradable model amino sugar substrate providing both organic carbon and nitrogen to stimulate primary fermentation processes and subsequent processes that are trophically linked to methanogenesis in Puukkosuo fen (Northern Finland), a nitrogen-limited, subarctic, meso-eutrophic and pH-neutral peatland dominated by *Carex* sedges and *Sphagnum* mosses (Palmer and Horn, 2015). The stimulatory effect of methanogenic substrates was evaluated to investigate potential bottle necks for the methanogens inhabiting the fen soil. In addition to soil incubation experiments, *in situ* measurements of CO_2_ and CH_4_ emissions as well as microbial community analysis of field fresh soil were performed to unravel bacterial taxa, methanogens and H_2_-metabolizers present at Puukkosuo fen (Supplementary Figure S1).

## Materials and methods

### Sampling site and soil parameters

The study site, Puukkosuo fen, is located in northeastern Finland (66°22′38″N, 29°18′28″E; elevation 200 m NN). The mean annual air temperature and mean annual precipitation at the site are −0.43 ± 0.09°C and 772 ± 12 mm, respectively (average of years 1966–2011, measured at Oulanka research station). Puukkosuo fen is pH-neutral (pH ~ 6.9), meso-eutrophic and water saturated with a vegetation consisting mainly of mosses (*Sphagnum* spp.) and graminoids (e.g., *Carex* spp.). Four replicate soil cores from layers 0 to 20 cm were taken on July 28th, 2010. These four cores were pooled on site and mixed thoroughly. The mixed sample was divided into subsamples for incubation studies and molecular analyses, and the subsamples were transported on ice to the laboratory where they were stored at 4°C for up to 11 months for microcosm analyses or at −80°C for approximately 3 weeks for nucleic acid extractions. The sampled surface soil had a soil moisture content of 90%, soil carbon, soil nitrogen and the carbon-to-nitrogen ratio were 434 g kg_DW_^−1^, 29 g/kg_DW_^−1^ and 15, respectively (Palmer and Horn, 2015).

### Assessment of *in situ* gas emissions

*In situ* gas emissions of fen soil were determined by the closed-chamber technique as described previously (Palmer and Horn, 2015). Briefly, closed poly(methyl methacrylate) (PMMA) chambers were placed onto the soil surface and gas samples (5 mL per sampling timepoint) were taken from the chambers via gas outlets at 4 timepoints (0–3 h). Prior to installation of the chambers, plots were watered with 2 L fen porewater without supplements (control) and with 20 mM supplemented ammonium (4 replicate plots per treatment). Gas samples were injected into gas tight evacuated containers (Exetainers, Labco Limited, High Wycombe, UK), and CO_2_ and CH_4_ concentrations were determined using Hewlett-Packard 5980 series II gas chromatographs equipped with thermal conductivity and flame ionization detectors, respectively, as described in Küsel and Drake (1995).

### Assessment of fermentation and methanogenesis potentials in soil microcosms

Two separate sets of anoxic microcosms experiments were conducted to assess fermentative and methanogenic potentials of the sampled surface soil (0–20 cm depth), respectively. All incubations were set up in triplicates of 1:10 dilutions of fen soil (20 g soil +180 mL anoxic water or 10 g of soil +90 mL of anoxic water) in gas-tight 0.5 L infusion flasks (Müller & Krempel, Bülach, Switzerland) that were sealed with screw caps and rubber stoppers (Glasgerätebau Ochs, Bovenden, Germany). Headspaces were flushed with sterile argon. For the fermentation potential experiments, the following treatments were set up in May 2011 and incubated at 20°C in the dark for 10 days: (i) unsupplemented control microcosms, (ii) microcosms supplemented with 500 μM N-acetylglucosamine (NAG), (iii) microcosms with 500 μM NAG and bromoethanesulfonate (BES; 20 mM), and (iv) unsupplemented control microcosms with BES. In the latter two microcosms, BES was used as an inhibitor for methanogenesis (Oremland and Capone, 1988), thus allowing for the detection of fermentation products that might be used by methanogens in microcosms without BES. Fermentation potentials were tested without preincubation of the microcosms. Net turnover of carbon and reductant for the time frames day 0–4, 4–10, and 0–10 was calculated as follows: The initial concentrations of compounds measured at the beginning of a time frame were subtracted from final concentrations measured at the end of a time frame in both, NAG treatments and unsupplemented controls. Then, the resulting concentrations in unsupplemented controls with and without BES were subtracted from those in NAG supplemented microcosms with and without BES, respectively, to get net turnover concentrations for each compound. Finally, net turnover concentrations of compounds were multiplied with 8/32 for NAG, 1/2 for formate, 2/12 for ethanol, 2/8 for acetate, 3/14 for propionate, 1/8 for methane, and 1/0 for CO_2_ to get net turnovers of carbon/reductant for each compound. Positive and negative values indicate net production and consumption of a compound in a given time frame, respectively.

For the methanogenic potential experiments, one subset of microcosms was set up in May 2011 and preincubated for 120 days at 20°C in the dark, and a second subset of microcosms was set up in July 2011 without preincubation. The preincubation served to reduce potentially existing alternative electron acceptors in the soil and thus stimulate peat methanogens. 1 mM formate, 1 mM acetate, 1 mM methanol or 8 vol % H_2_ (i.e., 8.4 mmol L^−1^ microcosm slurry) and 2 vol % CO_2_ (i.e., 2.1 mmol L^−1^ microcosm slurry) were supplemented at the beginning of the main incubation, which was 11 days at 20°C in the dark. Methanogenic substrates were resupplemented after 8 days of incubation (note that formate was not resupplemented in preincubated microcosms initially supplemented with formate). In addition to microcosms supplemented with potential methanogenic substrates, unsupplemented control microcosms with or without 20 mM BES were set up (note that BES was added at the beginning of the preincubation for the respective control of the preincubated microcosms).

Microcosms’ headspaces and liquid phases were sampled in 1–2 day intervals. Concentrations of CH_4_ and CO_2_ in gas samples were analyzed with a gas chromatograph (Hewlett Packard 5890 series II equipped with FID or TCD detector); reliable concentrations of H_2_ could not be quantified due to technical problems; concentrations of sugars, organic acids, and alcohols in liquid samples were analyzed via high pressure liquid chromatography equipped with an Aminex HPX-87H ion exclusion column and refractive index and UV detectors (Küsel and Drake, 1995). Amounts of gaseous and dissolved compounds in microcosms are given as μM concentrations and can be converted to μmol per g dry weight of soil by dividing with a conversion factor of 10.5.

### Molecular characterization of fen bacteria, hydrogen metabolizers, and methanogens

Nucleic acids were extracted from a single replicate of fresh homogenized pooled surface layer fen soil as previously described using a bead-beating protocol (Peršoh et al., 2008; Palmer and Horn, 2015). Bacterial 16S rRNA genes as well as the functional gene markers *mcrA,* [FeFe]-hydrogenase and group 4 [NiFe]-hydrogenase genes were amplified using the primer pairs 341F (5′-CCT ACG GGA GGC AGC AG-3′)/907RM (5′-CCG TCA ATT CMT TTG AGT TT-3′) (Muyzer et al., 1993), ME1 (5′-GCM ATG CAR ATH GGW ATG TC-3′)/ME2 (5′-TCA TKG CRT AGT TDG GRT AGT-3′) (Hales et al., 1996), NiFe-gF (5′-GAY CGI RTI TGY GGI ATY TGY GG-3′)/NiFe-gR (5′-GTR CAI GAR TAR CAI GGR TC-3′), and HydH1f (5′-TIA CIT SIT GYW SYC CIG SHT GG-3′)/HydH3r (5′-CAI CCI YMI GGR CAI SNC AT-3′) (Schmidt et al., 2010, 2011), respectively. Each primer was preceded by a 6-bp-long barcode (5′-ACTATC - gene specific primer-3′), to allow for separation of target sequences after batch sequencing as the sequencing run also included samples that were not part of the present study. Barcoded amplicon pyrosequencing of PCR products was conducted in 2011 as previously described at the Göttingen Genomics Laboratory using the Roche GS-FLX 454/Titanium technology (Palmer et al., 2012; Palmer and Horn, 2015). Sequence analysis of forward reads was done at a later date in QIIME 2 version 2022.2.0 (Bolyen et al., 2019). Imported sequences were demultiplexed using the q2-cutadapt plugin version 2022.2.0 by searching for the barcode + primer with a maximum error rate of 0.1 and removing them as a well as any preceding bases (Martin, 2011).

Quality controlled amplicon sequence variants (ASVs) were generated using the q-dada2 plugin for 454 sequencing (“denoise-pyro”; Callahan et al., 2016) with the following parameters: Sequences were trimmed to a length of 300 bp (“—p-trunc-len” 300), excluding any shorter sequences, and the “consensus” method was used for chimera detection and removal. Taxonomic assignment of ASVs was conducted by subjecting ASVs representative sequences of 16S rRNA gene and functional gene amplicons to a BLAST search of nucleotide (BlastN) and *in silico* translated protein sequences (BlastX), respectively, to identify the most closely related cultured relatives for each sequence (Altschul et al., 1990). Alpha diversity metrics [observed ASVs, Faith phylogenetic diversity (Faith, 1992), Shannon diversity (Shannon, 1948)] were calculated for reads subsampled at different sequencing depths using the “alpha-rarefaction” command of the q2-diversity plugin version 2022.2.0 in QIIME 2. Amplicon sequence reads have been deposited in the European Nucleotide Archive (ENA) under accession number PRJEB58427.

## Results

### *In situ* CO_2_ and CH_4_ emissions

**Figure 1:**
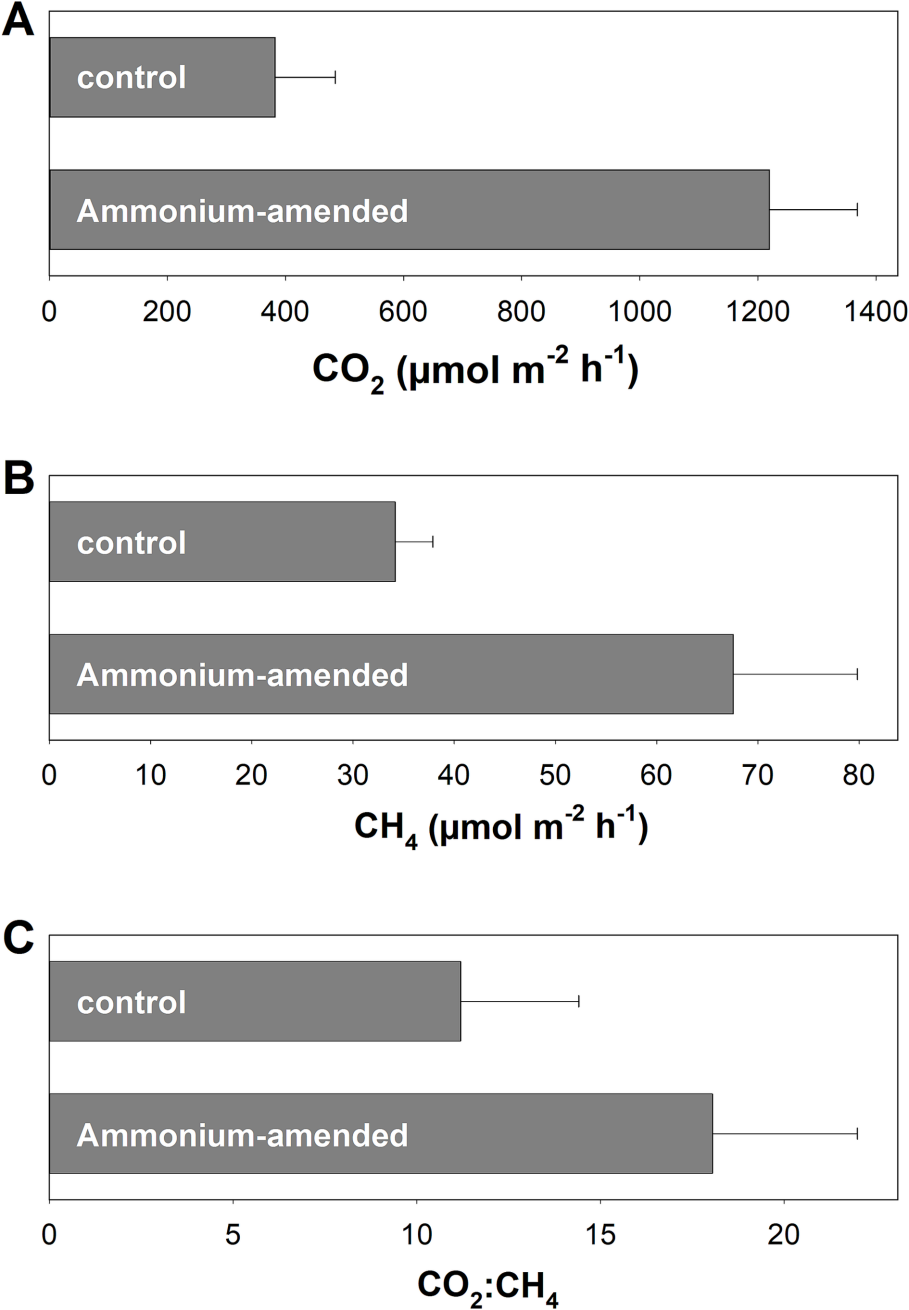
*In situ* emission of CO_2_ **(A)** and CH_4_ **(B)** as well as CO_2_:CH_4_ ratios **(C)** in control plots and plots amended with 2 L of fen porewater containing 20 mM ammonium. Measurements were conducted in August 2010. Mean values and standard errors of 4 replicates per treatment are displayed.

In Puukkosuo fen, CO_2_ and CH_4_ emissions of plots irrigated with unamended porewater were approx. 400 μmol m^−2^ h^−1^ and 35 μmol m^−2^ h^−1^, respectively (Figures 1A,B). Amendment with ammonium affected CO_2_ and CH_4_ emissions: Both CO_2_ and CH_4_ emissions were higher from plots with ammonium amendment, indicating that ammonium might stimulate methanogenesis as well as general microbial activity via alleviation of nitrogen limitation. The resulting CO_2_:CH_4_ ratios were approx. 12.5 and 18 for unamended and ammonium amended plots, respectively (Figure 1C).

### Phylogenetic and functional diversity of microorganisms in field-fresh fen soil

**Figure 2:**
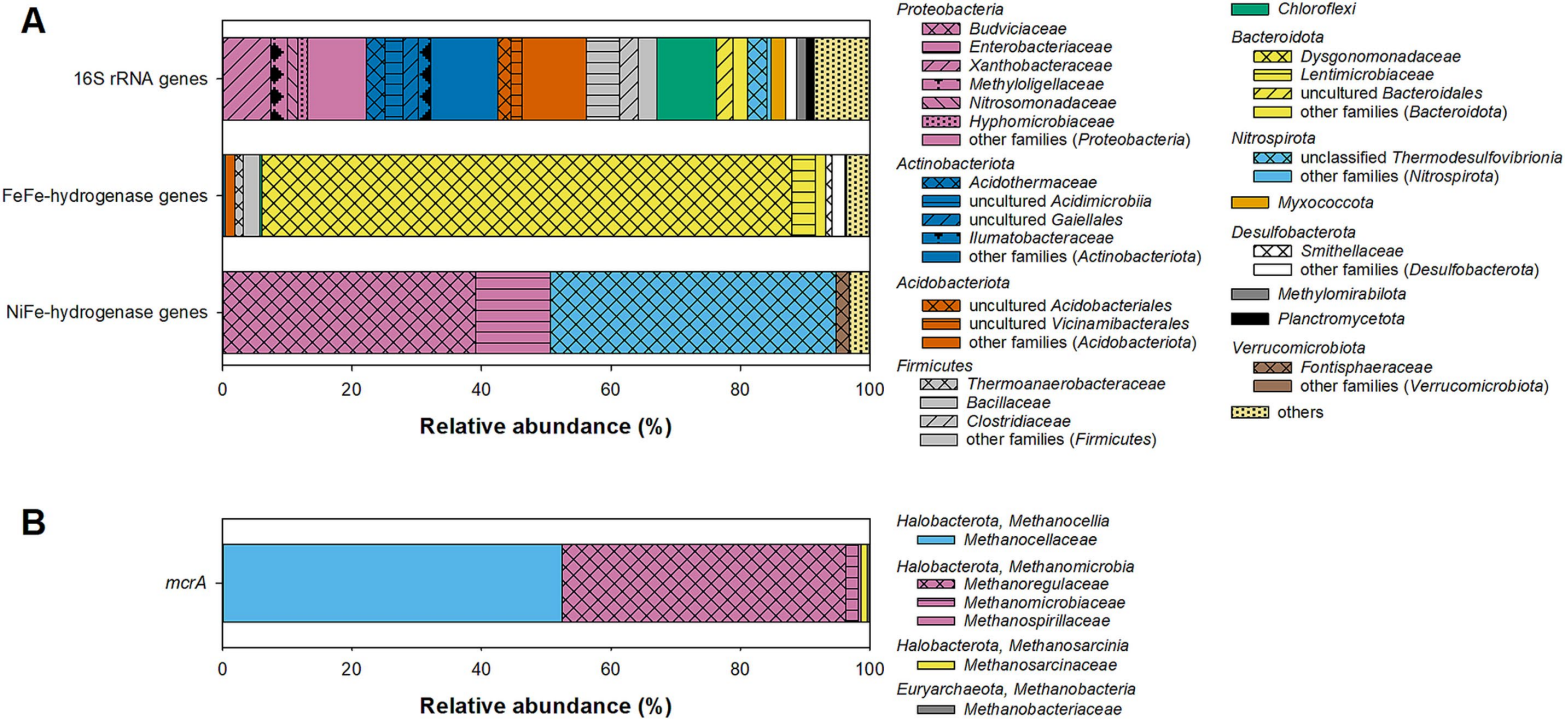
Relative abundance of bacterial **(A)** and archaeal **(B)** taxa in forward reads of **(A)** 16S rRNA genes, [FeFe]-hydrogenase genes, and group 4 [NiFe]-hydrogenase genes and **(B)** *mcrA* genes in field-fresh pH-neutral fen soil. Taxonomic classification was done on family-level based on the classifications in Supplementary Table S1. Families with relative abundances <1.5% were grouped as “other families (phylum).” Phyla with a relative abundance <1% and unclassified sequences were grouped as “others” in **(A)**.

Amplicon pyrosequencing of bacterial 16S rRNA genes yielded a total of 3,459 quality filtered reads forming 239 ASVs. Alpha diversity parameters indicated that the sequencing depth was sufficient to cover most of the bacterial diversity present in the fen (Supplementary Figure S2). Blast search revealed low (<90%) identity of some ASV representatives to sequences of cultured representatives (Supplementary Table S1), indicating that there is a high degree of uncultured bacterial diversity in Puukkosuo fen. The bacterial community was dominated by *Proteobacteria*, *Actinobacteriota, Acidobacteria* and *Firmicutes* (Figure 2A), microbial taxa that are commonly found to be abundant in peatlands (Pankratov et al., 2005; Dedysh, 2011; Tveit et al., 2013). Some of the detected ASVs were affiliated to genera known to comprise fermenters (e.g., *Clostridium*, *Pelosinus*, *Opitutus,* and *Spirochaeta*), acetogens (*Clostridium*), iron reducers (e.g., *Aciditherimonas*, *Anaeromyxobacter*, and *Geobacter*), and sulfate reducers (e.g., *Desulfosporosinus* and *Desulfomonile*) (Supplementary Table S1).

Nine hundred and eighty-one quality-filtered [FeFe]-hydrogenase reads were obtained which grouped into 58 ASVs (see Supplementary Figure S3 for alpha diversity plots). Identities of ASV representative sequences to sequences of the next cultured representatives ranged between 56 and 89% (Supplementary Table S1). Based on a previously established family-level hydrogenase amino acid sequence similarity cut-off of 80% (Schmidt et al., 2011), 82% of the [FeFe]-hydrogenase reads could be affiliated to the *Dysgonomonadaceae*, a family harboring propionate fermenters that can degrade carbohydrates and/or proteins (Hahnke et al., 2016) (Figure 2A). Other [FeFe]-hydrogenase ASVs with low relative abundance had identities above or next to 80% to known syntrophic fermenters (*Syntrophus* and *Smithella*), sulfate reducers (*Desulfovirgula*), and iron reducers (*Mesoterricola*) (de Bok et al., 2001; Kaksonen et al., 2007; James et al., 2016; Itoh et al., 2023) (Supplementary Table S1). Several ASVs were only distantly related (<70% identity) to hydrogenases of cultured bacteria (Supplementary Table S1), and an adequate phylogenetic or functional affiliation of such essentially novel [FeFe]-hydrogenases is not possible.

Two hundred and sixty quality-filtered reads were obtained for group 4 [NiFe]-hydrogenase genes, and it is likely that such low sequencing depth was insufficient to capture the full diversity of group 4 [NiFe]-hydrogenase genes. However, flattening out rarefaction curves indicated that the 20 ASVs that were detected largely represent the hydrogenase diversity that could be amplified with the used primer pair, which was originally designed to detect group 4 [NiFe]-hydrogenases of *Gammaproteobacteria* (Schmidt et al., 2011) (Supplementary Figure S4). Identities of ASV representative sequences to sequences of the next cultured representatives ranged from 68 to 86% (Supplementary Table S1). About one half of the group 4 [NiFe]-hydrogenase reads were affiliated to the *Enterobacterales*-families *Budviciaceae* and *Enterobacteriaceae* that harbor facultative aerobes performing mixed acid fermentation under anoxic conditions (Brenner and Farmer, 2005; Adeolu et al., 2016) (Figure 2A). Most of the remaining group 4 [NiFe]-hydrogenase reads formed three ASVs with 71% identity to the H_2_-oxidizing sulfate reducer *Thermodesulfovibrio thiophilus* (Supplementary Table S1); however, due to the relatively low identity, a closer phylogenetic and functional affiliation of the detected hydrogenases remains elusive.

For *mcrA*, 1,661 quality-filtered reads were obtained, which grouped into 37 ASVs (see Supplementary Figure S5 for alpha diversity plots). Most of the *mcrA* reads were affiliated either to the *Methanoregulaceae* (44% relative abundance) or the *Methanocellaceae* (52% relative abundance; Figure 2B). Both families comprise hydrogenotrophic methanogens that can be commonly found in peatlands or other wetlands (Oren, 2014a; Sakai et al., 2014). Notably, only one ASV (mcrA-fw-33; 1% relative abundance) was affiliated with the *Methanosarcinaceae*, a family that comprise many aceticlastic methanogens (note that the closest cultured relative, *Methanosarcina lacustris* (81% identity), does not utilize acetate as a growth substrate) (Oren, 2014b). None of the ASVs was affiliated with *Methanothrix* (formerly *Methanosaeta*), a genus comprising exclusively aceticlastic methanogens that have been repeatedly detected in peatlands (Kotsyurbenko et al., 2019; Bräuer et al., 2020). However, the used primer pair ME1/2 has been reported to be unsuitable for the amplification of *Methanothrix* (e.g., Lueders et al., 2001; Shigematsu et al., 2004), which needs to be taken into consideration.

### Product profiles in unsupplemented fen microcosms with and without BES addition

**Figure 3:**
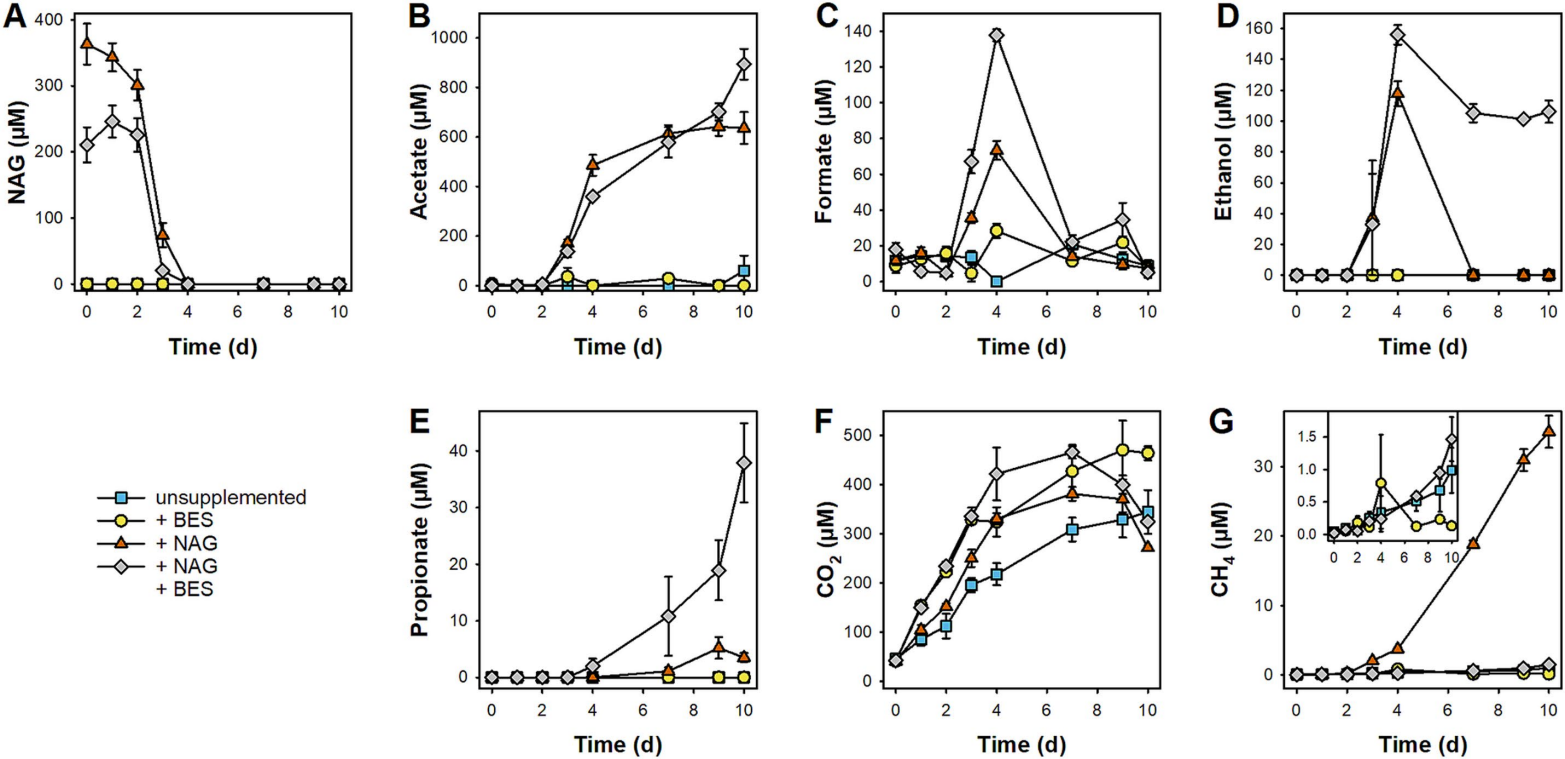
Effect of N-Acetylglucosamine (NAG) on production and consumption of fermentation products and methane in pH-neutral fen soil during 10 days of anoxic incubation. Microcosms were incubated in absence and presence of 20 mM BES (+ BES) as well as without and with supplemented NAG (+ NAG). Mean values of three replicates and standard errors are displayed for concentrations of NAG **(A)**, acetate **(B)**, formate **(C)**, ethanol **(D)**, propionate **(E)**, CO_2_ **(F)**, and CH_4_ **(G)**. The insert in panel **(G)** is an enlargement of the low concentration range to better show low levels of CH_4_.

In unsupplemented microcosms without BES, CO_2_ steadily accumulated, and it was by far the most prominent of the detected products (Figure 3). CH_4_ concentrations increased steadily as well, but comparatively low amounts of the gas accumulated during the 10 days of anoxic incubation (Insert in Figure 3G). The relatively low accumulation of CH_4_ compared to that of CO_2_ is reflected by generally high CO_2_:CH_4_ ratios that were highest at day 2 (1,600) and subsequently decreased to ~300 toward the end of the incubation (Supplementary Figure S6). Thus, while methanogenesis became relatively more important over time, alternative (and unresolved) respiratory processes dominated the mineralization of endogenous peat organic carbon. Other fermentation products frequently detected in anoxic peat microcosms like acetate, formate, ethanol, and propionate did not accumulate (Figures 3B–E). In unsupplemented microcosms with added BES, CH_4_ accumulation was suppressed while the residual product profile was overall similar to that in unsupplemented microcosms without BES.

### Effect of supplemental NAG on the product profiles in fen microcosms with or without BES

In NAG-supplemented microcosms without BES, NAG was consumed completely within 4 days without appreciable delay, with most rapid consumption from day 2 to 4 (Figure 3A). NAG consumption was accompanied by the accumulation of acetate, formate, ethanol, and CO_2_, suggesting ongoing primary fermentation (Figure 3). In the second stage of the incubation (Day 4–10), formate, ethanol, and partially CO_2_ were consumed while mainly CH_4_, some acetate, and traces of propionate accumulated. In NAG-supplemented microcosms with BES, the same fermentation products were observed as in NAG-supplemented microcosms without BES during the initial stage (Day 0–4), however, transient accumulation of formate and ethanol were more prominent with BES (Figure 3). In the subsequent stage (Day 4–10), concentrations of formate, ethanol, and CO_2_ decreased, whereas acetate and some propionate accumulated in NAG-supplemented microcosms with BES, in which methanogenesis was largely blocked.

**Figure 4:**
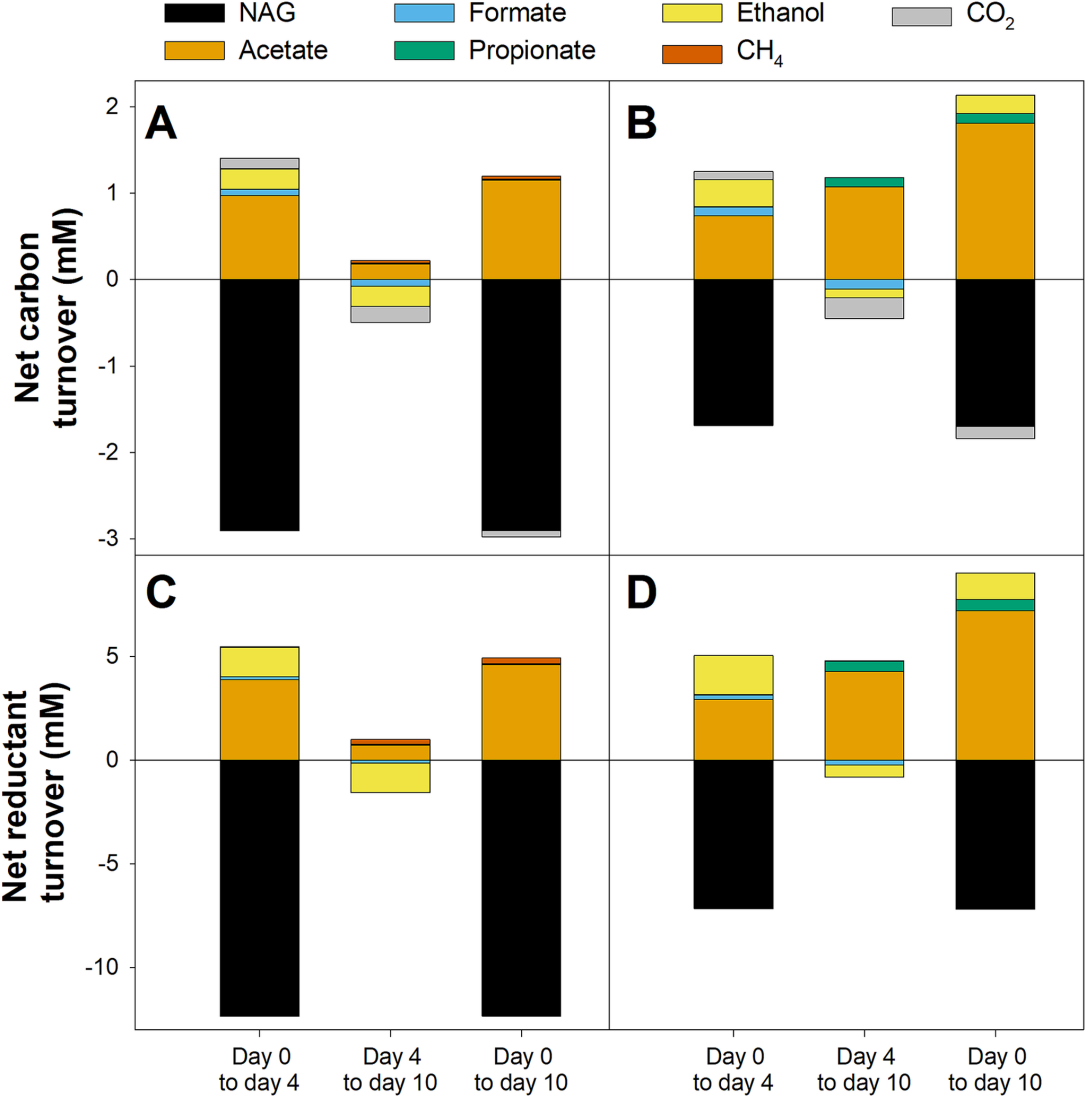
Net turnover of carbon **(A,B)** and reductant **(C,D)** for different stages in N-Acetylglucosamine (NAG) supplemented anoxic fen soil microcosms. Microcosms were supplemented with NAG and incubated in absence **(A,C)** or presence of 20 mM BES **(B,D)**. See the materials and methods section for a detailed description of the calculation. Mean values of three replicates are displayed.

Net carbon and reductant turnover analysis was performed to determine which of the observed products could be directly linked to NAG consumption and which products were formed during the degradation of primary NAG-fermentation products. In NAG-supplemented microcosms without BES, only about 50% of the carbon and reductant theoretically derived from the amount of NAG consumed could be recovered in the detected products during the initial stage (Day 0–4, Figures 4A,C). Acetate accounted for ~75% of recovered carbon and reductant and was therefore the dominant detected fermentation product followed by ethanol, CO_2_, and formate. During the second stage (Day 4–10, Figures 4A,C), net consumption of ethanol, formate, and CO_2_ could account for the net production of acetate and CH_4_. Carbon and reductant recovered in acetate, ethanol, formate, and CO_2_ accounted for most of the NAG that was degraded during the initial incubation in NAG-supplemented microcosms with BES (Day 0–4, Figures 4B,D). In the subsequent stage (Day 4–10, Figures 4B,D), only a part of the acetate and propionate that were formed could be explained by the observed consumption of ethanol, formate, and CO_2_, indicating that other undetected sources of carbon and reductant were converted as well. Furthermore, when comparing the net carbon and reductant turnover in NAG-supplemented microcosms without BES to that in NAG-supplemented microcosms with BES over the whole 10 day incubation period, it was remarkable that less than half the consumed carbon and reductant could be recovered in the absence of BES (Day 0–10) whereas more carbon and reductant were recovered in the products than what was theoretically available from the substrates in the presence of BES (Figure 4). The reasons for this discrepancy were unknown.

### Effect of methanogenic substrates on CH_4_ production in fen soil microcosms with and without preincubation

**Figure 5:**
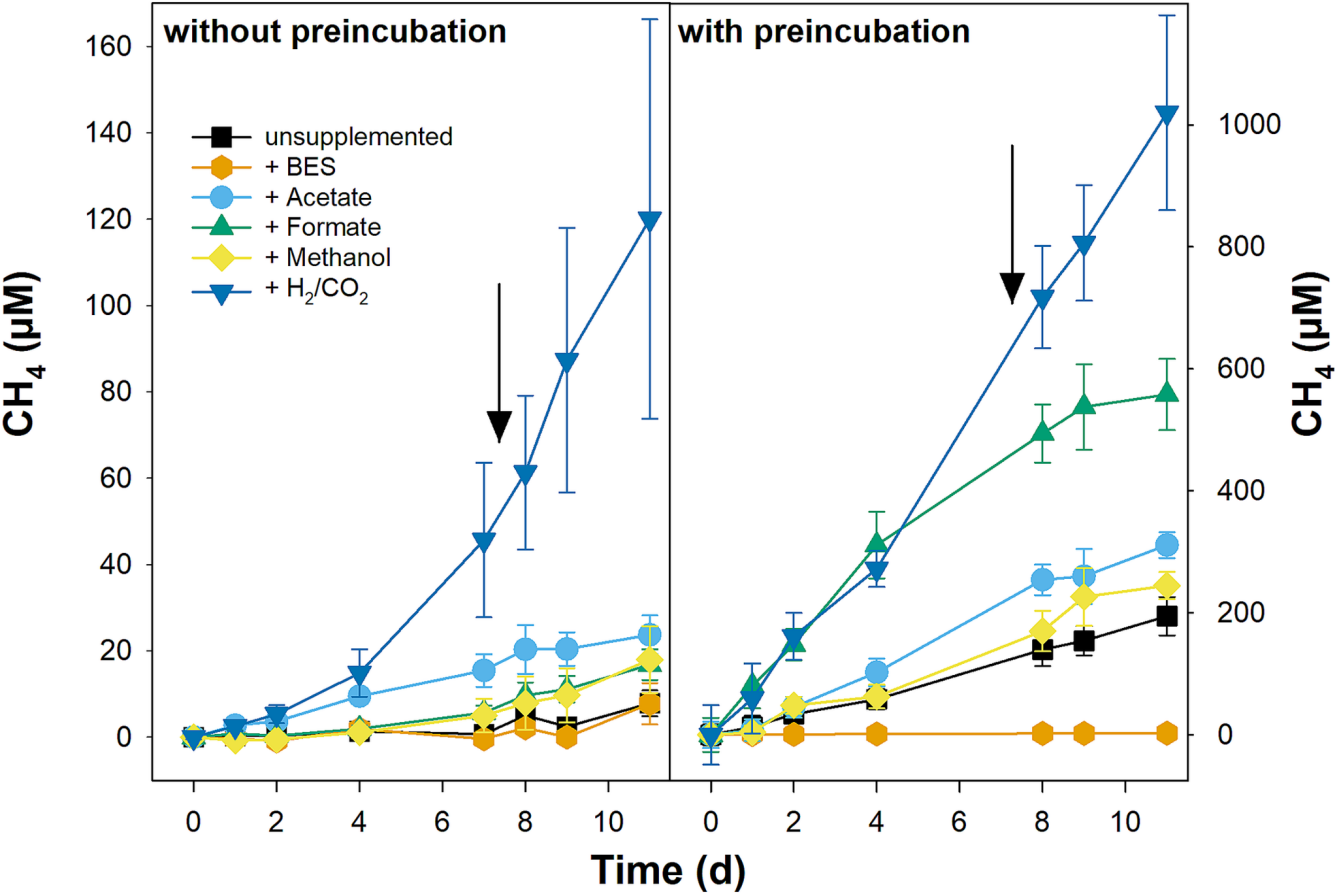
Effect of methanogenic substrates on the production of CH_4_ in pH-neutral fen soil. Microcosms were set-up without (left) and with (right) preincubation of 120 days to reduce alternative electron acceptors. Microcosm were left unsupplemented or supplemented with 20 mM BES, 1,000 μM acetate, formate, or methanol, or with 8% vol. H_2_ and 2% vol. CO_2_. Substrates were resupplemented after sampling on day 7 (indicated by arrows; formate was not resupplemented in the preincubated microcosms). Mean values of three replicates and standard errors are displayed. Methane present at t_0_ was subtracted to allow for better comparison of net CH_4_ production in microcosms without and with preincubations.

CH_4_ accumulation in unsupplemented microcosms without preincubation was slow (Figure 5). An equally slow CH_4_ accumulation was observed in unsupplemented microcosms of the fermentation experiment that were also not preincubated (Figure 3). In contrast, CH_4_ accumulated readily and without delay in unsupplemented microcosms preincubated for 120 d in which the final CH_4_ concentrations were more than 30 times higher than in unsupplemented microcosms without preincubation (Figure 5). All tested supplemented substrates (H_2_/CO_2_, formate, acetate, and methanol) stimulated CH_4_ production in microcosms with and without preincubation; however, the stimulatory effect was more pronounced in preincubated microcosms (Figure 5). In fact, the fast initial consumption of supplemental formate and supplemental acetate in microcosms without preincubation was largely uncoupled from CH_4_ accumulation, indicating that these substrates were predominately consumed by microbial oxidation processes others than methanogenesis at least at the start of the incubation (Figure 5; Supplementary Figure S7). In preincubated microcosms, the stimulatory effect on CH_4_ accumulation was highest for H_2_/CO_2_ and formate and lowest for methanol (Figure 5). Note that, in contrast to the other substrates, formate was not resupplemented in preincubated microcosms (Supplementary Figure S7), and therefore CH_4_ accumulation slowed down once the initially supplemented formate was consumed (Figure 5). Supplemental H_2_/CO_2_ did not only stimulate methane accumulation but also the accumulation of acetate, especially in microcosms without preincubation (Supplementary Figure S7), indicating that hydrogenotrophic methanogenesis and hydrogenotrophic acetogenesis were going on in parallel. Acetate also accumulated in BES-supplemented microcosms without preincubation and during the preincubation in BES-supplemented microcosms with preincubation in which methanogenesis was largely blocked (Figure 5; Supplementary Figure S7). Whether acetate accumulation in BES-supplemented microcosms was due to ongoing acetogenesis or fermentation could not be resolved. However, the accumulation of propionate, either during the incubation or during the preincubation, indicates that fermentation processes were going on in BES-supplemented microcosms (Supplementary Figure S7).

In summary, the stimulatory effect of methanogenic substrates could be better evaluated in microcosms with 120 d preincubation in which electron acceptors other than CO_2_ were presumably largely depleted. The potential for hydrogenotrophic methanogenesis was higher than for aceticlastic methanogenesis, while the potential for methylotrophic methanogenesis was low during the 11 d incubation.

## Discussion

### Puukkosuo fen, a pH-neutral peatland as source for atmospheric CH_4_

To date, peatlands are well recognized for their complex importance for the global climate since they can function as sinks or sources for the greenhouse gasses CO_2_, CH_4_, and N_2_O (Frolking and Roulet, 2007; Strack et al., 2008; Harris et al., 2022). Previously, the potential to function as N_2_O sink has been reported for the pH-neutral Puukkosuo fen (Palmer and Horn, 2015). In the present study, *in situ* gas measurements at Puukkosuo fen showed that CH_4_ was emitted alongside CO_2_, indicating that at the time point of the measurements methanogenesis was ongoing and exceeded or bypassed methane oxidation (Figure 1B). The 35 μmol CH_4_ m^−2^ h^−1^ that have been emitted on average were in the lower range of what has been observed in other peatlands (Horn et al., 2003; Hamberger et al., 2008; Salmon et al., 2022). However, the values given in the aforementioned references are maximum emissions for the respective sites measured over a much longer period than in the present study, and prolonged measurement campaigns would be needed to determine the temporal changes and the range in CH_4_ emissions at Puukkosuo fen over different seasons and years. The finding that *in situ* CH_4_ emissions were higher when ammonium was amended indicated that methanogenesis was limited by N-availability (Figure 1B), which is in line with the low concentrations of inorganic nitrogen reported for the fen (Palmer and Horn, 2015). Puukkosuo fen is a pristine peatland and anthropogenic N-influx or -deposition is likely limited. Thus, methanogens and other microbes rely on natural reactive nitrogen input like precipitation and groundwater flow, energy intensive nitrogen fixation, and/or the release of nitrogen from the decomposition of peat organic matter.

### Primary fermentation activities

As discussed above, the overall microbial activity and methanogenesis in particular are limited by the availability of nitrogen in Puukkosuo fen. Therefore, NAG as an easily degradable carbon and nitrogen source was chosen as model substrate to study primary fermentation and subsequent degradation processes in peat soil microcosms. The immediate fermentation of NAG indicates that fen microbes are poised to consume NAG (Figure 3). Acetate, ethanol and formate were the primary fermentation products that were observed during NAG consumption in the presence and absence of BES (Figures 3, 4). The fermentation profile is indicative of mixed acid fermentation as performed by facultative aerobic enterobacteria (Müller, 2008) which were abundant in group 4 [NiFe]-hydrogenase gene libraries of fresh peat soil (families *Budviciaceae* and *Enterobacteriaceae* in Figure 2). However, the detected fermentation products can be also produced by other fermentative taxa like many members of the *Clostridiaceae* (Wiegel, 2015), a family that accounted for 3% of the 16S rRNA gene sequences retrieved from fresh peat soil (Figure 2). A time-resolved community analysis on transcript or protein level would be necessary to better identify active fermenters in NAG-supplemented peat soil microcosms.

Acetate and ethanol were also observed in NAG-supplemented microcosms with peat soil from a temperate, moderately acidic fen (Wüst et al., 2009). While acetate is also commonly observed as major fermentation product in peat soil microcosms supplemented with glucose or xylose, ethanol was not detected so frequently when these sugar monomers were fermented (Kotsyurbenko et al., 1996; Hamberger et al., 2008; Wüst et al., 2009; Hunger et al., 2015). Nevertheless, ethanol was detected in fresh and incubated peat soil and was a potential product of the fermentation of root-derived organic carbon (Metje and Frenzel, 2005, 2007; Tveit et al., 2015; Meier et al., 2021). Butyrate and propionate are other fermentation products that can transiently or permanently accumulate in sugar-supplemented peat soil microcosms (Kotsyurbenko et al., 1996; Hamberger et al., 2008; Wüst et al., 2009; Hunger et al., 2015). However, propionate accumulation was largely uncoupled from NAG consumption and butyrate was not detected in the present study (Figure 3). Interestingly, butyrate was observed during NAG fermentation when the initial concentrations of the substrate were increased to 0.9 mM or higher (i.e., at least twice as high as in the present study), suggesting a tight coupling of butyrate production and consumption in the fen (Wüst et al., 2009).

While propionate accumulation was not observed in unsupplemented microcosms with BES (without preincubation) in the NAG experiment (Figure 3E), propionate accumulated after day 7 in the respective treatment of the methanogenic substrates experiment (Supplementary Figure S7). In addition, propionate accumulated during the preincubation of microcosms with BES (Supplementary Figure S7). These findings indicated that propionate was produced during the fermentation of endogenous organic matter derived from Puukkosuo fen soil. In this regard, (transient) propionate accumulation was observed in microcosms with unsupplemented peat soil before (Schmidt et al., 2015; Tveit et al., 2015), suggesting that propionate is a common product of the fermentation of peat-derived organic carbon. Taxa known to comprise propionate fermenters dominated in [FeFe]-hydrogenase gene libraries (*Dysgonomonadaceae* and *Lentimicrobiaceae* (Chen and Dong, 2005; Hahnke et al., 2016; Sun et al., 2016), Figure 2) and were also present in 16S rRNA gene libraries (ASV r85, 97.3% identity to *Psychrosinus fermentans* (Sattley et al., 2008), and ASV r238, 98.3% identity to *Opitutus* sp. VeSm13 (Janssen et al., 1997); Supplementary Table S1) of fresh Puukkosuo fen soil.

### Secondary fermentation activities

The term secondary fermenter is mostly used when describing a syntrophic association in which an alcohol- or organic acid-oxidizing bacterium (i.e., the secondary fermenter) depends on H_2_ (or formate) removal by a methanogenic partner (Schink, 1997). However, ethanol can be converted to propionate and acetate independent of interspecies hydrogen transfer between a fermenter and a methanogen (Schink et al., 1987), and such a conversion of ethanol was proposed to occur in anoxic incubations with arctic, pH-neutral peat soil (Tveit et al., 2015). In the current study, ethanol consumption in parallel to propionate accumulation was observed especially in the second stage (between day 4 and day 10) of NAG treatments with BES in which methanogenesis was inhibited (Figure 3). Propionate accumulation was less pronounced in NAG treatments without BES in which the transiently accumulated ethanol was completely consumed between day 4 and day 7 (Figure 3). This suggested that ethanol oxidation was largely performed in syntrophic association without the formation of propionate [as known for *Pelobacter carbinolicus* (Schmidt et al., 2014)] when methanogenesis was not inhibited. Alternatively, syntrophic oxidation of propionate might have prevented the accumulation of propionate derived from ethanol oxidation in NAG treatments without BES. In this respect, [FeFe]-hydrogenase genes affiliated with the family *Smithellaceae* were detected in fresh peat soil (Figure 2) and the type species of this family, *Smithella propionica,* is capable of syntrophic propionate oxidation (de Bok et al., 2001). Syntrophic propionate oxidation likely also prevented the accumulation of propionate derived from the fermentation of endogenous organic carbon in unsupplemented microcosms with or without preincubation (Supplementary Figure S7). Syntrophic propionate oxidation was studied in a moderately acidic fen in Germany in which *Smithella* and *Syntrophobacter*, two genera with contrasting metabolic pathways of propionate oxidation, were identified as active propionate oxidizers (Schmidt et al., 2016). However, the responsible propionate oxidizers in the neutral Puukkosuo fen still need to be identified.

### Methanogenesis

Hydrogenotrophic methanogens are well known partners of syntrophic fermenters. *mcrA*-gene libraries of fresh peat soil were largely dominated by ASVs that were affiliated to either *Methanoregulaceae* or *Methanocellaceae* (Figure 2), both of which comprise hydrogenotrophic methanogens (Oren, 2014a; Sakai et al., 2014). Both families together represent the dominant groups of hydrogenotrophic methanogens in various peatlands (Galand et al., 2002, 2005; Hunger et al., 2011, 2015; Yavitt et al., 2012; Blake et al., 2015; Schmidt et al., 2016; Bräuer et al., 2020). In line with the high abundance of hydrogenotrophic methanogens in fresh Puukkosuo fen soil, CH_4_ production was highest in H_2_/CO_2_-supplemented microcosms with or without preincubation (Figure 5). Except for *Methanoregula boonei*, all other cultured members of the *Methanoregulaceae* and *Methanocellaceae* can utilize formate in addition to H_2_ as electron donor during methanogenesis (Oren, 2014a; Sakai et al., 2014). Thus, it was not surprising that supplemental formate stimulated CH_4_ production equally well as supplemental H_2_/CO_2_ until formate got depleted in microcosms with preincubation (Figure 5). Interestingly, the stimulatory effect of formate was much weaker compared to that of H_2_/CO_2_ in microcosms without preincubation although formate was available throughout the incubation (Figure 5; Supplementary Figure S7). The lower availability of acetate might have caused weaker methane production in formate-supplemented microcosms without preincubation compared to those supplemented with H_2_/CO_2_ (Supplementary Figure S7), since all cultured members of the *Methanoregulacea* and *Methanocellaceae* require acetate for growth (Oren, 2014a; Sakai et al., 2014).

While supplemental H_2_/CO_2_ and formate commonly stimulate methanogenesis in peat microcosms, supplemental acetate can have a positive, neutral or negative effect (Williams and Crawford, 1984; Kotsyurbenko et al., 1996; Horn et al., 2003; Bräuer et al., 2004; Wüst et al., 2009; Hunger et al., 2011, 2015; Blake et al., 2015). The inhibitory effect of acetate on methanogens is observed exclusively in acidic peatlands in which undissociated acetic acid permeating into the cell can be toxic to methanogens at elevated concentrations (Russell, 1991; Horn et al., 2003; Bräuer et al., 2004). A stimulatory effect of supplemental acetate on CH_4_ production, as observed in the present study (Figure 5) and in other peatlands (Bräuer et al., 2004; Hunger et al., 2015), indicates that the potential of acetate consumption by the complex microbial community involved in methanogenic degradation exceeds the acetate production from endogenous organic carbon. As discussed above, the stimulatory effect of acetate in the present study could be explained by the acetate requirements for growth of *Methanoregulaceae* and *Methanocellaceae*, the two dominant methanogenic taxa observed in fresh peat soil (Figure 2) (Oren, 2014a; Sakai et al., 2014). In addition, acetate might have been converted to CH_4_ either in syntrophic cooperation between acetate oxidizers and hydrogenotrophic methanogens (Nusslein et al., 2001; Hattori, 2008) or by aceticlastic methanogens. It must be pointed out that aceticlastic methanogens of the family *Methanosarcinaceae* were largely absent in the *mcrA*-gene libraries of field fresh fen soil (Figure 2). However, in a slightly alkaline fen in Germany *Methanosarcinaceae* were not detected in field fresh soil, but represented 4–27% of the *mcrA*-gene sequences after 3 weeks of incubation in microcosms with contrasting substrate supply (see Mire 1 in Hunger et al., 2015), underlining that rare aceticlastic methanogens can considerably increase in relative abundance within comparatively short incubation times. Furthermore, the relative abundance of aceticlastic *Methanotrichaceae* in field fresh soil could not be evaluated due to the insufficient coverage of their *mcrA* genes by the primer set ME1/2 (Lueders et al., 2001; Shigematsu et al., 2004).

Methanol, which is released by living plants and the degradation of plant material (Galbally and Kirstine, 2002), is conceptualized to be only of minor importance as a substrate for methanogens in peatlands (Drake et al., 2009; Kotsyurbenko et al., 2019). Nevertheless, methanol can have a stimulatory effect on methanogenesis in peat soil microcosms (Bräuer et al., 2004; Wüst et al., 2009; Jiang et al., 2010). In the present study, a relatively weak stimulation of methanogenesis was observed in Methanol-supplemented microcosms with and without preincubation (Figure 5), suggesting that methanol is probably not a major substrate for methanogenesis in Puukkosuo fen.

### Acetogenesis

The ecological significance of acetogens in peatlands is still uncertain despite recent findings that indicated the contribution of acetogens to acetate production in some peatlands (Drake et al., 2009; Hunger et al., 2011, 2015; Hädrich et al., 2012; Ye et al., 2014; Kotsyurbenko et al., 2019; Meier et al., 2022). In the 16S rRNA gene library of field fresh peat soil, ASV r72 (0.8% relative abundance) and ASV r73 (0.3% relative abundance) were affiliated with the two acetogens *Clostridium magnum* (98.3% identity) and *Clostridium muellerianum* (94.3% identity), respectively (Supplementary Table S1) (Bomar et al., 1991; Doyle et al., 2022). In this respect, distinct acetate accumulation in H_2_/CO_2_-supplemented microcosms without preincubation and to a lesser extent in H_2_/CO_2_-supplemented microcosms with preincubation as well as in formate-supplemented microcosms with and without preincubation suggested that acetogens successfully competed with methanogens for both substrates under the experimental conditions (Supplementary Figure S7). Similarly, acetate accumulation was observed in H_2_/CO_2_ or formate supplemented microcosms in various peatlands, suggesting that acetogens successfully compete with methanogens at elevated concentrations of these substrates (Kotsyurbenko et al., 1996; Horn et al., 2003; Bräuer et al., 2004; Wüst et al., 2009; Hunger et al., 2011, 2015, 2016; Hädrich et al., 2012; Meier et al., 2022). Some acetate accumulated in methanol-supplemented microcosms with or without preincubation (Supplementary Figure S7), and it is possible that the weak stimulatory effect of methanol on methanogenesis was due to trophic links between acetogens and methanogens rather than direct consumption of methanol by methanogens (Jiang et al., 2010). Furthermore, ethanol can be a substrate for acetogens (Bertsch et al., 2016), and the finding that ethanol was consumed parallel to acetate accumulation during the second stage of the NAG-supplemented microcosms with or without BES might indicate that acetogens were involved in the degradation of NAG-derived ethanol (Figures 3, 4). In this regard, it has been hypothesized that acetogens could be involved in the degradation of ethanol released during the fermentation of root organic carbon in peatlands (Meier et al., 2021, 2022).

As discussed above, syntrophic acetate oxidation to CH_4_ might play a role in Puukkosuo fen. In such a trophic cooperation acetate conversion to H_2_/CO_2_ or formate by the acetate oxidizer can be conducted via the oxidative citric acid cycle or the oxidative acetyl-CoA cycle (Müller et al., 2015; Manzoor et al., 2016). The latter resembles the reversal of H_2_/CO_2_- or formate-dependent acetogenesis (Zinder, 1994), and some acetogens can indeed reverse their metabolism according to the thermodynamic conditions and substrate availability (Lee and Zinder, 1988; Oehler et al., 2012). However, if acetogens function as acetate oxidizers when H_2_ concentrations are low and acetogenesis becomes thermodynamically unfavorable in Puukkosuo fen remains to be elucidated.

### Alternative respiratory processes

In theory, CO_2_ and CH_4_ are produced in a 1:1 ratio during the complete anaerobic degradation of organic carbon at the redox level of glucose if methanogenesis is the sole terminal electron accepting process (C_6_H_12_O_6_ → 3CO_2_ + 3CH_4_) (Zinder, 1993), whereas ratios >1 are usually attributed to the use of other inorganic electron acceptors like O_2_, nitrate, ferric iron, or sulfate in addition to CO_2_ (McCarty, 1972). Humic substances that are abundant in peat can be used as organic electron acceptors, and thus also contribute to high CO_2_:CH_4_ ratios in peat soil *in situ* or *in vitro* (Heitmann et al., 2007; Roden et al., 2010; Gao et al., 2019). In this study, average CO_2_:CH_4_ ratios measured *in situ* were 12.4 ± 3.1 (Figure 1C), suggesting that methanogenesis was not the sole terminal electron accepting process in Puukkosuo fen at the time point of sampling. In this respect, a high denitrification potential has been observed in Puukkosuo fen although the availability of nitrate is low (Palmer and Horn, 2015). Notable sulfate concentrations were detected in the soil (53 and 23 μg/gDW in 0–20 cm and 20–40 cm soil, respectively), and such a relatively small pool of sulfate can sustain comparatively high rates of sulfate reduction when reduced sulfur compounds are continuously reoxidized (Pester et al., 2012). 16S rRNA gene and [FeFe]-hydrogenase gene ASVs related to sulfate reducers of the genera *Desulfosporosinus*, *Desulfomonile*, and *Desulfovirgula* of fresh peat soil underlined the possibility of sulfate reducers contributing to the anaerobic degradation of organic matter in Puukkosuo fen (Supplementary Table S1). Furthermore, some 16S rRNA gene ASVs were related to iron reducers from the genera *Aciditherimonas*, *Anaeromyxobacter*, and *Geobacter* (Supplementary Table S1), indicating that iron reduction, which can be important in peatlands (Reiche et al., 2008), might play a role in anaerobic degradation of organic carbon in Puukkosuo fen. Moreover, aerobic or anaerobic methane oxidation might have contributed to CO_2_:CH_4_ ratios greater than 1 as well (Smemo and Yavitt, 2011; Zalman et al., 2018). In this respect, ASV r154 and ASV r229 of the 16S rRNA gene library were affiliated to the methanotrophic genera *Methylocystis* and *Methylobacter* (Supplementary Table S1).

CO_2_:CH_4_ ratios in unsupplemented fresh peat soil microcosms were much higher than those observed *in situ* (Figure 1C; Supplementary Figure S6), supporting the assumption that sample handling, storage, and incubation procedures can obscure methanogenic conditions when peat soil is incubated *in vitro*, supporting the need for a pre-incubation to better mimic *in situ* near conditions (Yavitt and Seidman-Zager, 2006).

## Conclusions and future perspectives

The accumulation of fermentation products in NAG supplemented microcosms indicated a high fermentation potential of the microbial community in the fen, and formate as well as ethanol as important intermediates (Figure 3). Such fermentations were performed by a fen community harboring phylogenetically novel hydrogenases and organisms, thus the fen represents a reservoir of hitherto unknown microbial diversity.

In the present study, NAG, a monomer of the second most abundant biopolymer chitin, was used as a model compound. Since polymer hydrolysis often is the rate-limiting step resulting in low concentrations of NAG, chitin supplementation, and treatments with NAG in the lower μM range with refeeding coupled to time-resolved mRNA analysis of peat soil microcosms is recommended to identify key organisms of chitin and NAG degradation. Future research should likewise address the fate of acetate at Puukkosuo fen as a model system for pH neutral subarctic fens.

In conclusion, the microbial community of the CH_4_-emitting Puukkosuo fen showed a pronounced response to supplemental substrates for fermentation and hydrogenotrophic methanogenesis, indicating a high potential activity of both processes. Elucidating active primary and secondary fermenters, the role of acetogens, as well as the pathways of anaerobic acetate conversion to methane and involved taxa are key challenges for future research. Our current study provides a good starting point for conducting such subsequent studies.

## Data Availability

The datasets presented in this study can be found in online repositories. The names of the repository/repositories and accession number(s) can be found below: https://www.ebi.ac.uk/ena, PRJEB58427.
