# Supplementary material for: Synergy and competition during the anaerobic degradation of N-acetylglucosamine in a methane-emitting, subarctic, pH-neutral fen
Source: Front Microbiol. 2024 Dec 11;15:1428517. doi: 10.3389/fmicb.2024.1428517 (PMC11670324; doi:10.3389/fmicb.2024.1428517)
Supplement: Supplementary file 1 [file Presentation_1.pptx]

## Slide 1
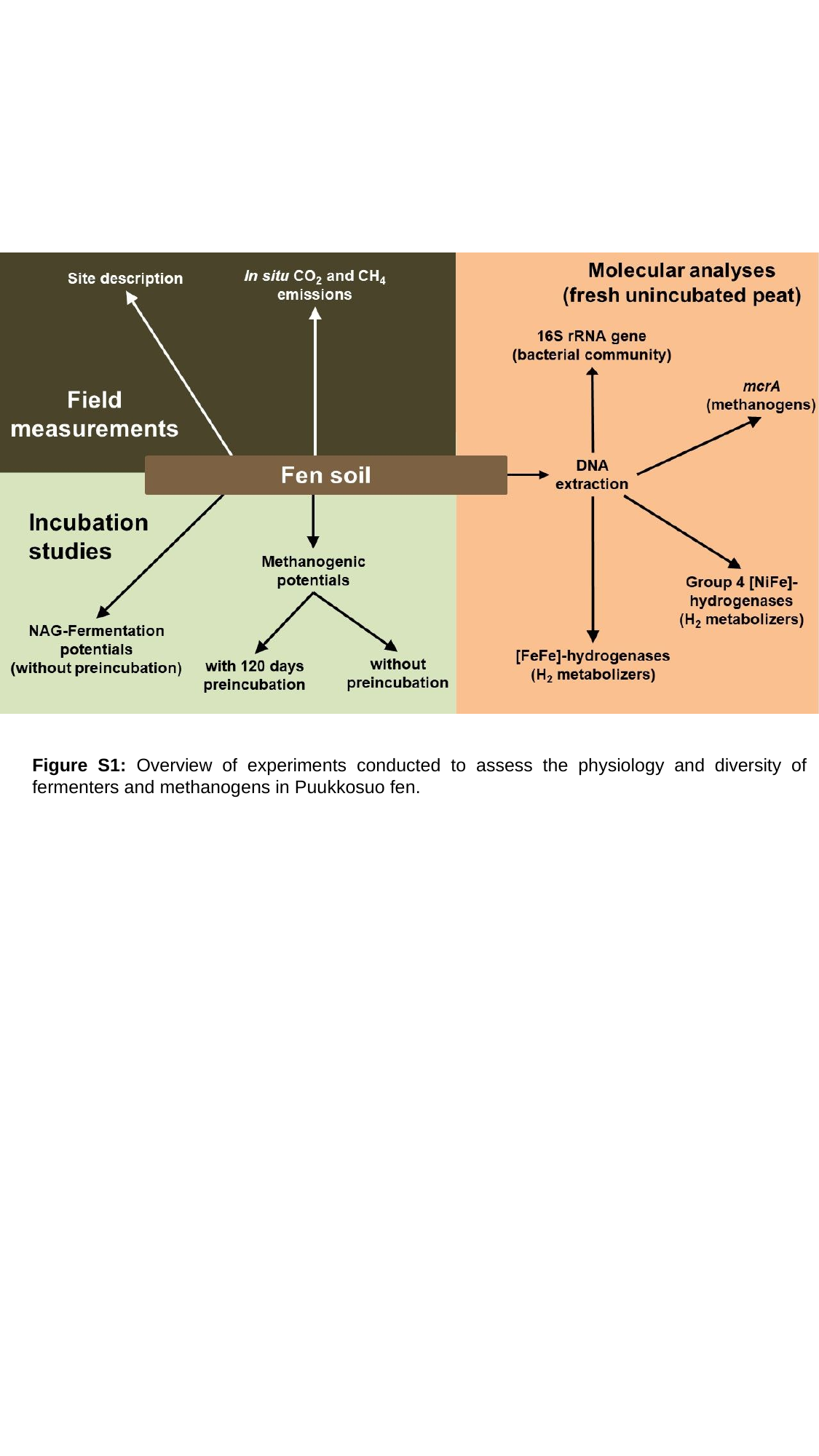

Figure S1: Overview of experiments conducted to assess the physiology and diversity of fermenters and methanogens in Puukkosuo fen.

## Slide 2
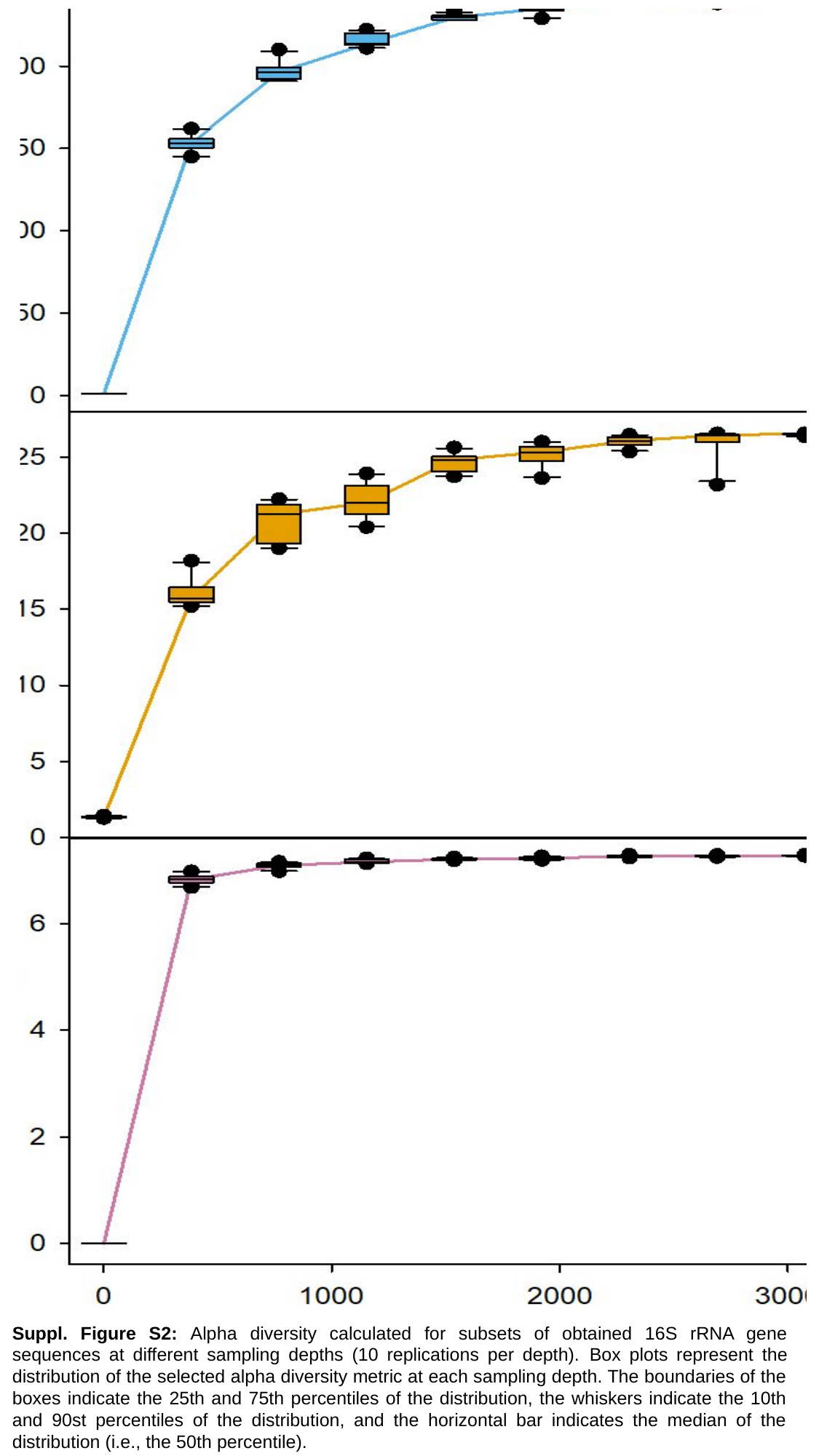

Suppl. Figure S2: Alpha diversity calculated for subsets of obtained 16S rRNA gene sequences at different sampling depths (10 replications per depth). Box plots represent the distribution of the selected alpha diversity metric at each sampling depth. The boundaries of the boxes indicate the 25th and 75th percentiles of the distribution, the whiskers indicate the 10th and 90st percentiles of the distribution, and the horizontal bar indicates the median of the distribution (i.e., the 50th percentile).

## Slide 3
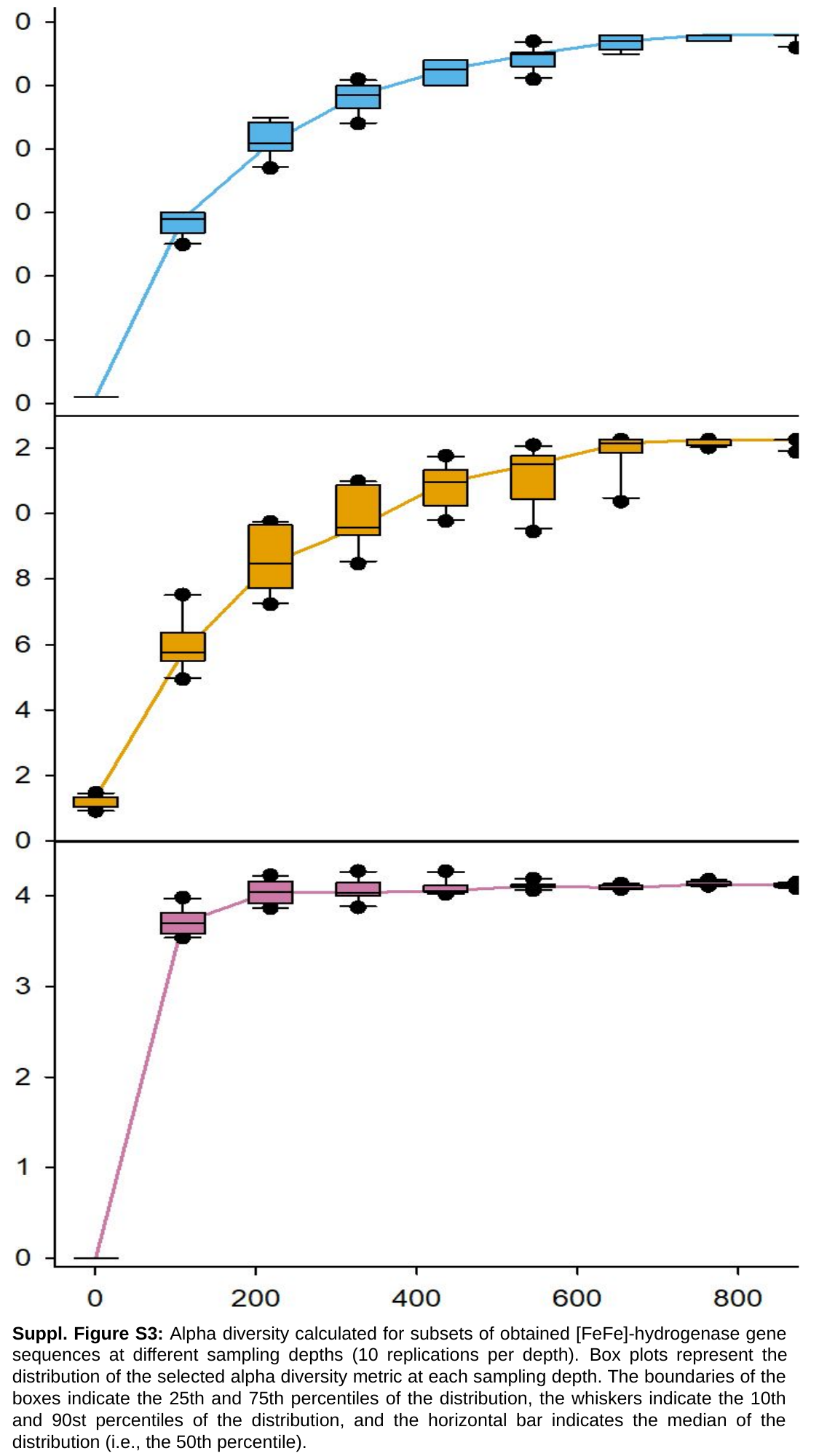

Suppl. Figure S3: Alpha diversity calculated for subsets of obtained [FeFe]-hydrogenase gene sequences at different sampling depths (10 replications per depth). Box plots represent the distribution of the selected alpha diversity metric at each sampling depth. The boundaries of the boxes indicate the 25th and 75th percentiles of the distribution, the whiskers indicate the 10th and 90st percentiles of the distribution, and the horizontal bar indicates the median of the distribution (i.e., the 50th percentile).

## Slide 4
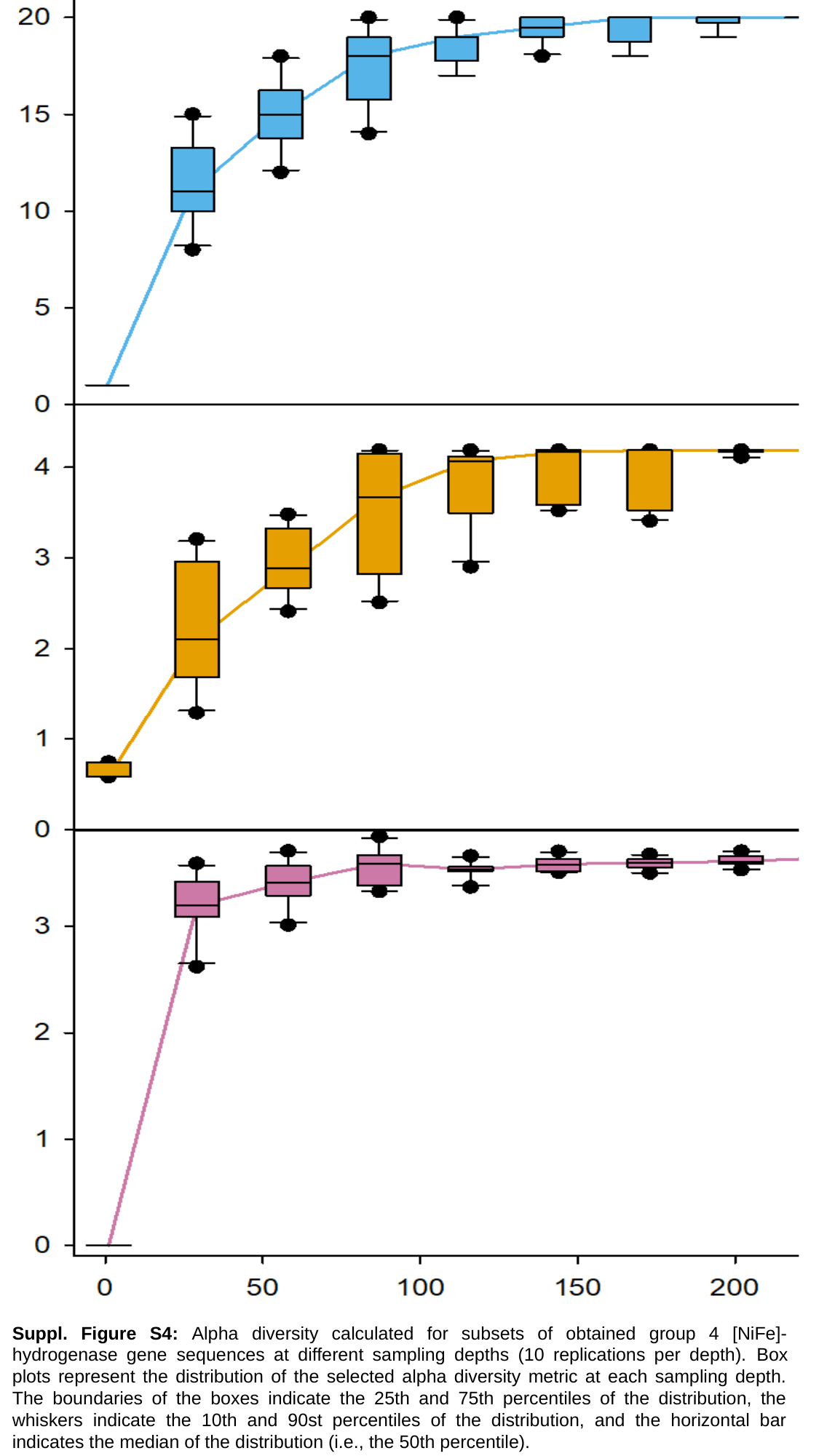

Suppl. Figure S4: Alpha diversity calculated for subsets of obtained group 4 [NiFe]-hydrogenase gene sequences at different sampling depths (10 replications per depth). Box plots represent the distribution of the selected alpha diversity metric at each sampling depth. The boundaries of the boxes indicate the 25th and 75th percentiles of the distribution, the whiskers indicate the 10th and 90st percentiles of the distribution, and the horizontal bar indicates the median of the distribution (i.e., the 50th percentile).

## Slide 5
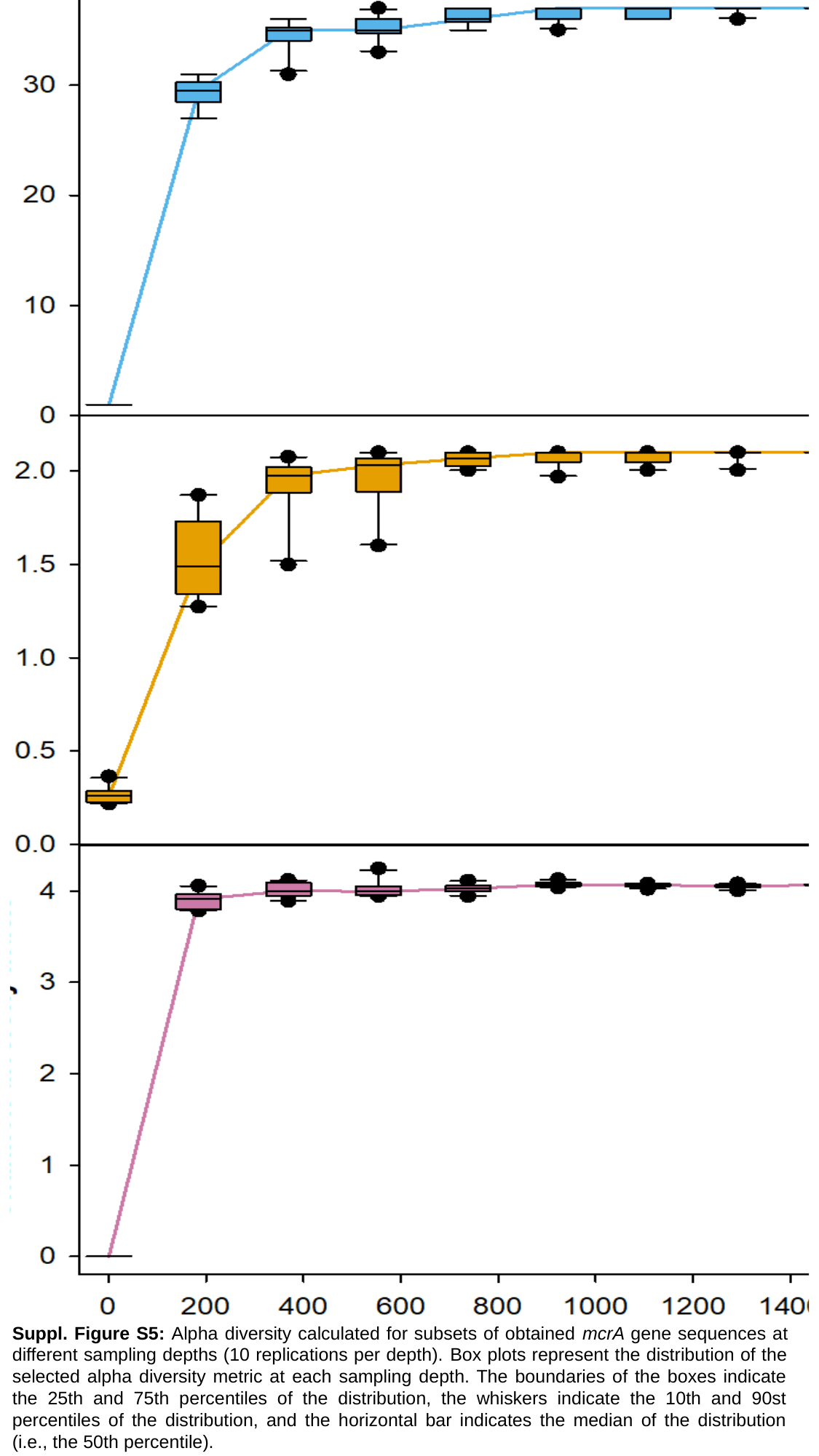

Suppl. Figure S5: Alpha diversity calculated for subsets of obtained mcrA gene sequences at different sampling depths (10 replications per depth). Box plots represent the distribution of the selected alpha diversity metric at each sampling depth. The boundaries of the boxes indicate the 25th and 75th percentiles of the distribution, the whiskers indicate the 10th and 90st percentiles of the distribution, and the horizontal bar indicates the median of the distribution (i.e., the 50th percentile).

## Slide 6
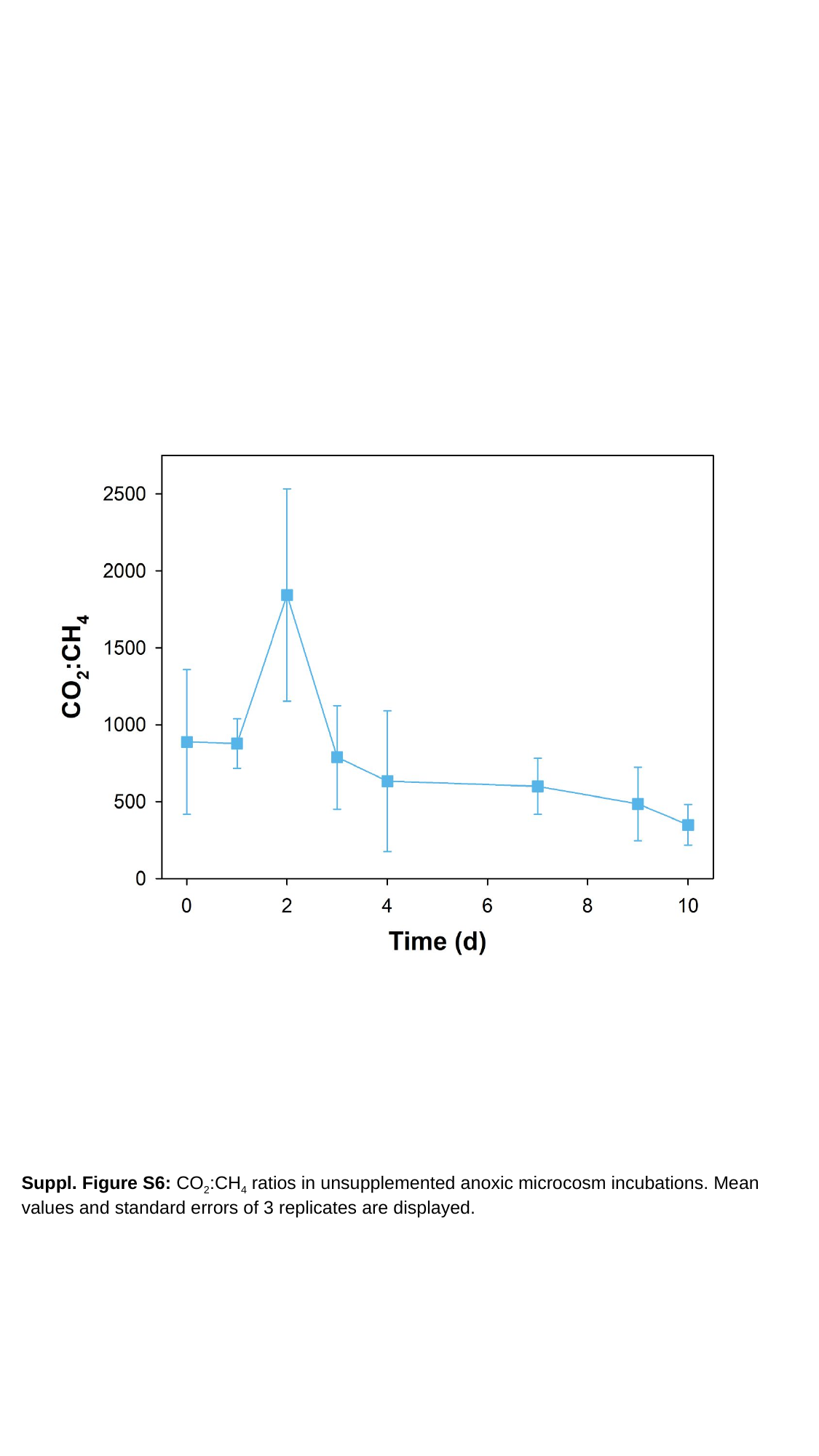

Suppl. Figure S6: CO2:CH4 ratios in unsupplemented anoxic microcosm incubations. Mean values and standard errors of 3 replicates are displayed.

## Slide 7
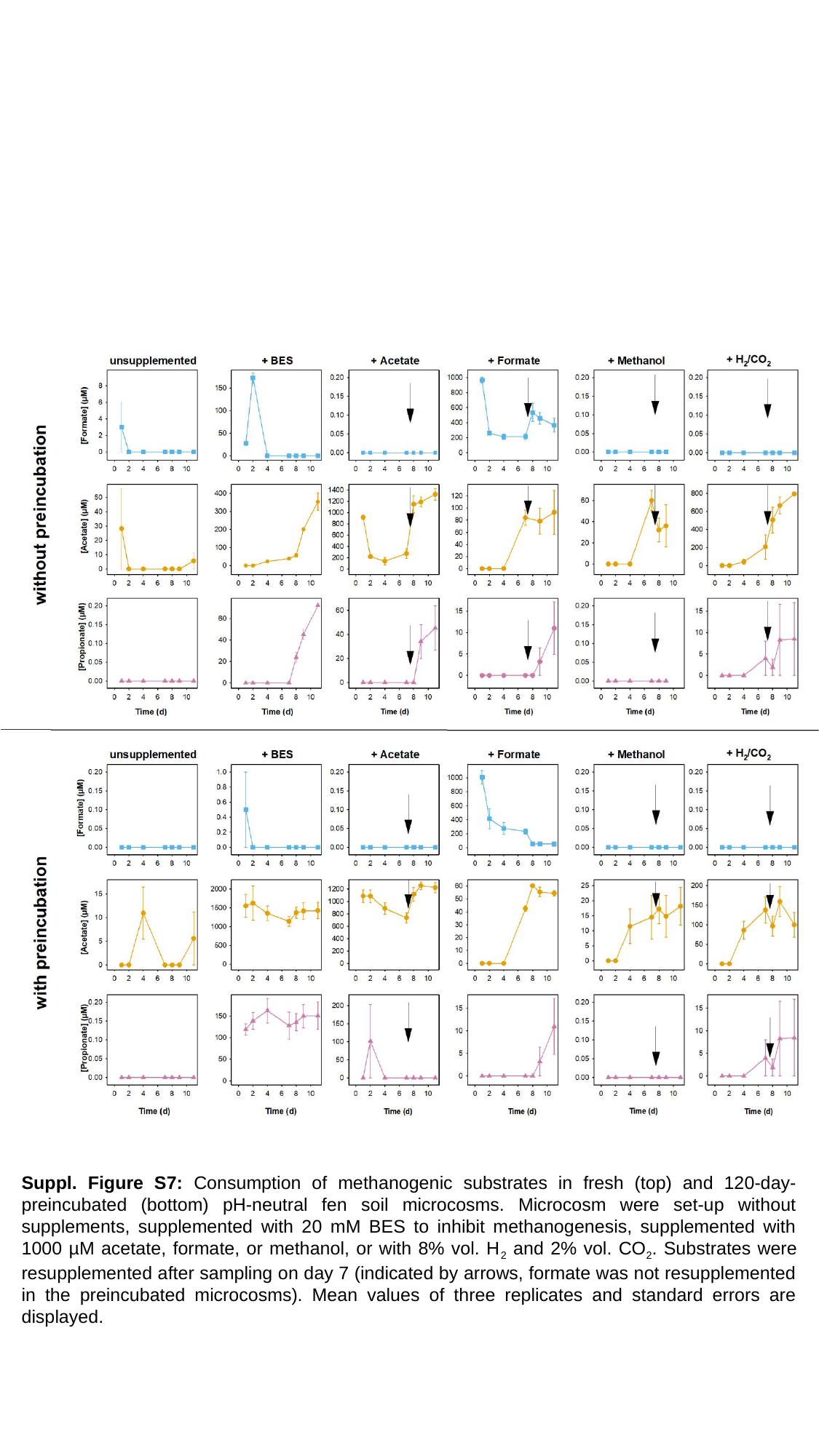

Suppl. Figure S7: Consumption of methanogenic substrates in fresh (top) and 120-day-preincubated (bottom) pH-neutral fen soil microcosms. Microcosm were set-up without supplements, supplemented with 20 mM BES to inhibit methanogenesis, supplemented with 1000 µM acetate, formate, or methanol, or with 8% vol. H2 and 2% vol. CO2. Substrates were resupplemented after sampling on day 7 (indicated by arrows, formate was not resupplemented in the preincubated microcosms). Mean values of three replicates and standard errors are displayed.
